# Analyzing exercise-to-rest ratios in U19 American football European championship: implications for team success and injury prevention

**DOI:** 10.3389/fspor.2024.1466118

**Published:** 2024-12-23

**Authors:** Valentin Prioul, Jean Slawinski, Steeve Guersent, Laure Le Monnier, Vincent Goeb, Florent Krim, Philippe Lopes, Pierre-Marie Leprêtre

**Affiliations:** ^1^ Faculty of Sciences and Techniques in Physical Activities and Sports, Research Unit of Physiological Adaptations to Exercise (APERE, EA-3300), University of Picardie Jules Verne, Amiens, France; ^2^ National Training Center and Talent Detection, French Federation of American Football, Amiens, France; ^3^ Expertise and Performance (INSEP), Research Unit Sport Exercise and Performance (SEP, EA-7370), French National Institute of Sport, Paris, France; ^4^ Rheumatology Department, University Hospital of Amiens—Picardie, Amiens, France; ^5^ Unit of Cardiac Rehabilitation, Hospital Center of Corbie, Corbie, France; ^6^ Laboratoire de Biologie de l'exercice pour la performance et la santé, Institut de Recherches Biomédicales des Armées, Paris Saclay, Evry, France; ^7^ Univ Rouen Normandie, Normandie Univ, CETAPS UR 3882, Boulevard Siegfried, 76821 Mont-Saint-Aignan, France

**Keywords:** team sport, performance, video analysis, young players, running-passing activity ratio, football

## Abstract

**Background:**

The teams' collective playing strategy rather than the individual player attitudes could explain event outcome and risk of injuries.

**Objective:**

The study aimed to examine the playing style of European teams and compare it to the USA.

**Method:**

12 matches from the U19 European championship of American Football were analysed. We characterized each team by their running-passing activities ratio during the offensive phase: running (RUN), passing (PASS), or balanced (BAL).

**Results:**

The BAL style of play accurately described 75% of European teams (*n* = 6). The remaining two national teams adopted either the RUN or PASS style of play. This finding is similar to what was observed in NCAA Division IA. All RUN style teams had a higher percentage of rushing plays (80.7% ± 9.7%) compared to PASS (33.0% ± 14.7%) and BAL teams (46.0 ± 0.8%) (*p* < 0.05). The mean playing time for RUN and PASS teams was longer than the average duration of plays for BAL teams (*p* < 0.05). The mean duration of plays ranged from 5.3 ± 1.9 to 5.7 ± 2.1 s, with a significant style of play effect (*p* < 0.05). Rest duration for BAL teams (46.7 ± 44.1 s) was shorter than that of RUN (55.9 ± 34.7 s) and PASS (54.5 ± 32.9 s) teams (*p* < 0.05). Finally, the European top final team was the team that was able to shift their game style during the tournament and presented a low coefficient of variation in offensive plays per drive.

**Conclusion:**

Based on the running-passing activities ratio, the video match analysis can provide a foundation for the strength and conditioning program for long-term athlete development and injury prevention.

## Introduction

The scientific literature reported that injury incidence was significantly higher in team compared with individual sports as a result of a higher incidence of both traumatic and overuse injuries (1). A sequence of steps in injury prevention model was proposed to prevent sport injuries (2, 3). Recently, Bolling et al. (3) recommended taking into account the practice forms right from the first step of the Van Mechelen Model of injury prevention. American football is the most popular sport to watch live in the United States of America, with the Super Bowl serving as the pinnacle of the sport (4). The 56th Super Bowl, which took place in 2022, was the most-watched show in five years in the United States, with a mean audience of 101.1 million TV viewers and 11.2 million streaming views (5). These statistics demonstrate the immense popularity of the sport, which extends beyond the borders of North American countries, as evidenced by the millions of streaming views. In fact, American football is played in 74 countries around the world, including 45 nations that have competitive championships at least (6). Due to the allowed contacts, American football has a reputation for being a violent sport with a very high risk of injury. Hence, studies in American football often focus on key areas to understand and reduce injuries (7). In fact, A study of Tator (8) highlighted that head trauma and spinal cord injuries are amor the most serious and common in American Football. Video recordings have typically been used to investigate situations involving concussion and injury occurrence during American football competitions (9–16). Current works focus primarily on shoulder injury patterns or concussions (17, 18). However, Mack et al. (19) showed that lower extremity injuries affect a high number of high level American Football players. With the hamstrings being the primary aero of concern (19), we can question the effects of the adopted collective strategy on the hamstrings’ overuse. Video-based analysis is also considered vital for achieving optimal performance (20). Coaches and sports scientists use video recordings to classify matches based on specific patterns or visual qualities that allow the categorization of match styles in team sports (21). This approach has been used to establish that the collective strategy of playing, rather than the individual player's attitude, may explain the outcome of the event. To the best of our knowledge, few studies have explored American football play styles neither to explain the outcome of the event nor the risk of injuries (12, 13, 16, 22–24). The style of the team's play during the offensive phase is typically defined by the ratio of running to passing activities (12, 13, 16, 23, 25). According to Iosia and Bishop (23), teams can be broadly classified into three categories based on their offensive style: rushing (RUN), passing (PASS), or balanced (BAL). A team with 69.0% or more of its plays being runs was considered to have a rushing style, while a team with less than 31.0% rushing plays was classified as having a passing style. Teams with a percentage of rushing or passing plays lower than 55.0% of total offensive plays were considered to have a balanced style (23). Iosia and Bishop (23) reported that there were significant differences in play duration and rest time between the RUN and PASS styles in the National Collegiate Athletic Association (NCAA) Division IA college football championship.

Studies have also demonstrated that the three different styles of play demand specific athletic skills (12, 13, 16, 23). Similarly, as with other team sports, video match analysis is useful for evaluating the players’ metabolic characteristics needed to implement team strategy and tactical formation (26, 27). Recently, Bayliff et al. (28) tracked athletes with a global positioning system to investigate the physical demands of American Football, but they did not consider the team's play style. Therefore, video match analysis based on the running-passing activities ratio could provide a basis for a strength and conditioning program for long-term athlete development. In this way, video analysis can be helpful for comparing the difference between player abilities and sport demands. Therefore, video match analysis based on the running-passing activities ratio could be useful for evaluating the mechanical deviations of the athlete from normal to identify improper stresses on the body that could predispose an athlete to injury, especially in young athletes at the development stage (29, 30). Therefore, it is crucial to exercise caution when drawing conclusions from the observations of the NCAA Division IA college football championship and applying them to other non-American championships. The NCAA Division IA is the pinnacle of college football in the United States and attracts the most talented student-athletes from around the world. Empirical observations suggest that the phases of games in the American championship are faster than in those of other nations. It is unclear whether this difference in game speed is due to technical-tactical aspects or physical qualities. At the scientific level, only a few authors have focused on comparing the physical qualities of U.S. players and non-American players (31, 32). Previous studies have reported significant differences in physical attributes and performance between players from Italy, Japan, and the United States (31, 32). To our knowledge, no study has attempted to explain these empirical observations by quantifying the style of play found in the different championships. This raises the question of whether the style of play and the skills of the players interact with each other and whether previous classifications of teams can be applied to American football events outside of the United States. Before the COVID-19 pandemic, six European national teams were ranked among the top 10 in the world. The objective of this study was to examine the effects of the style of play on the outcome of the U19 European championship. It was hypothesized that the style of play could be characterized by the ratio of running-passing activities and involve a comparison of European teams to a reference from the United States.

## Method

### Dataset

In 2019, the IFAF Under 19 European Championship football matches were held in Italy, bringing together the top eight European teams under the age of 19. From July 29th to August 4th, Austria, Denmark, Finland, France, Italy, Norway, Spain, and Sweden competed for the U19 title (Table 1). Each national team played three games, resulting in a total of 12 matches being recorded. To conduct our analysis, we extracted data from videos posted on the Italian Federation of American Football's YouTube channel, the IFAF website, YouTube®, and Facebook® social media platforms (12, 16, 22, 23, 25). This study was approved by the French Ethical Committee for Research in Sports, Physical Activities Sciences and Technologies (CERSTAPS) and registered under IRB00012476-2022-16-02-154.

**Table 1:** Style of offense delineation.

Team	Total plays	Run plays	Pass plays	Rushing (%)	Passing (%)	Style of play
Austria	168	98	70	58.3	41.7	BAL
Denmark	192	102	90	53.1	46.9	BAL
Finland	187	82	105	43.9	56.1	BAL
France	145	116	29	80.0	20.0	RUN
Italy	168	57	111	33.9	66.1	PASS
Norway	155	67	88	43.2	56.8	BAL
Spain	160	78	82	48.8	51.3	BAL
Sweden	156	75	78	48.1	50.0	BAL

RUN: 69.0% of offensive plays were runs at least, PASS: rushing plays were less than 31.0% of total offensive plays and BAL: rushing plays were lower than 55.0% of total offensive plays (23).

### Video analysis of selected game activities

The present study focused solely on offensive plays, excluding special teams such as punts, punt return, kick-off, and kick-off return. Each game was timed using a stopwatch, with a play-by-play sheet serving as the data collection tool, indicating all plays during the game. The duration of each play was determined by timing the game segments from the snap of the ball to the end of the play. The end of play was determined by the referee's whistle or an abrupt stop in the action, such as an incomplete pass, sack, or tackle that occurred before the referee could mark the progress and blow his whistle. Rest intervals between plays were calculated by timing the game segments from the whistle ending a play until the snap of the ball for the next play (22, 23). Plays were classified into three main categories: RUN, PASS or BAL. Therefore, the style of each team was defined by their offensive activities, as previously proposed (23). The style of play was determined by reviewing the global statistics of each national team during the tournament. Video analysis was carried out one trained experimenter familiar with the American football rules and activities.

### Statistical analysis

The data is presented as the mean ± standard deviation (min; max). Data were analyzed using JASP statistical software (version 0.16.0.0, University of Amsterdam, Amsterdam, The Netherlands) after being computed in a table file (Excel 16.16.27, Microsoft Corporation, Redmond, United States). The Levene's test was used to determine if variances were equal, and a Bonferroni's *post hoc* test was used to identify significant differences. Three one-way ANOVAs were used to analyze data in case of the equality of variances. Alternatively, Kruskal-Wallis test was applied for the comparison of rest time between plays, rest duration after a play based on rushing of the offense and after a play characterized by passing plays. The coefficient of variation was calculated based on the number of plays per drive. A conformity test was applied to compare the results obtained from European championship analysis with the previous study on the NCAA Division IA college football championship. The level of significance was set at *p* < 0.05.

## Results

During the course of the tournament, the average number of plays per match was 109.9 ± 8.9 (97; 124). The offensive strategies employed by each team are presented in Table 1. The final statistics for the tournament indicated that five of the eight teams (62.5%) utilized a balanced offensive style (BAL). These teams included the second-place team, Sweden, as well as those ranked from fourth to sixth, and the team in last place. The victorious Austrian team's offensive strategy was close to BAL, with 58.3% of their total offensive plays being rushing plays. The French team, which finished in third place, employed a rushing-based offensive strategy (RUN), while the Italian team that finished in seventh place adopted a passing-based offensive strategy (PASS). Table 2 displays the tournament averages, results, and distribution of drives, plays, and plays per drive, broken down by final ranking and offensive strategy. With the exception of France, all teams modified their offensive strategies throughout the tournament. The coefficient of variation for drives ranged from 4.7% in Sweden to 26.0% in France, and for plays, the range was from 7.2% in Italy to 19.9% in France. For plays-per-drive, the range was from 8.9% in Italy to 19.5% in France. Among all teams, the French team had the highest coefficient of variation for all three metrics. In contrast, there was no difference in drive, plays, and plays-per-drive between the top two teams, characterized by a BAL style of play, the RUN team in third place, the PASS team, and the others. There was no significant difference in RUN and PASS plays between the top three teams, Austria, Sweden, and France. However, the French team had a higher percentage of rushing plays (80.7% ± 9.7%) compared to Italy (33.0% ± 14.7%) and other EU teams (46.0% ± 0.8%) (*p* < 0.05). Additionally, there was a statistically significant difference for PASS plays, expressed as a percentage of the style of offense, between France and Italy (19.3% ± 9.7% vs. 66.0% ± 14.5%, *p* < 0.05).

**Table 2:** Game statistics during tournament.

Team		Austria	Sweden	France	Italia	Other countries
Final ranking		1	2	3	7	–
Tournament style of play		BAL	BAL	RUN	PASS	BAL
Event 1	Result	W	W	W	De	–
	Drive	10	13	10	13	11.3 ± 0.6
	Plays	53	61	53	54	54.3 ± 14.6
	Plays per drive	5.3 ± 1.8	4.8 ± 2.1	4.4 ± 2.9	4.3 ± 2.6	4.8 ± 1.0
	RUN	57	28	83	30	49.6 ± 6.4
	PASS	43	67	17	67	38.3 ± 25.5
	Style of play	BAL	PASS	RUN	PASS	BAL
Event 2	Result	W	W	De	Dr	–
	Drive	12	12	12	12	12.0 ± 1.0
	Plays	44	45	54	54	60.3 ± 8.4
	Plays per drive	4.1 ± 1.3	3.8 ± 1.9	4.5 ± 2.1	4.5 ± 2.1	4.9 ± 0.8
	RUN	52	60	70	20	45.4 ± 1.1
	PASS	59	40	30	80	52.1 ± 0.7
	Style of play	BAL	BAL	RUN	PASS	BAL
Event 3	Result	W	De	W	W	–
	Drive	11	12	7	10	12.0 ± 2.0
	Plays	51	50	37	61	55.5 ± 10.4
	Plays per drive	4.6 ± 2.3	4.2 ± 1.9	3.1 ± 3.6	5.1 ± 3.2	4.5 ± 0.8
	RUN	67	60	89	49	43.8 ± 8.8
	PASS	33	40	11	51	53.8 ± 8.6
	Style of play	RUN	BAL	RUN	BAL	BAL
Mean ± SD (CV)	Drive	11.0 ± 1.0 (9.1)	12.3 ± 0.6 (4.7)	9.6 ± 2.5 (26.0)	11.7 ± 1.5 (7.2)	11.8 ± 1.3 (11.2)
	Plays	49.3 ± 4.7 (9.6)	52.0 ± 8.2 (15.7)	48.0 ± 9.5 (19.9)	56.3 ± 4.0 (7.2)	56.6 ± 10.2 (18.1)
	Plays per drive	4.6 ± 0.5 (10.9)	4.6 ± 0.5 (11.8)	4.0 ± 19.5 (19.5)	4.6 ± 0.4 (8.9)	4.7 ± 0.8 (16.4)
	RUN	58.7 ± 7.6 (13.0)	49.3 ± 18.5 (37.4)	80.7 ± 9.7 (12.0)	33.0 ± 14.7^F^ (44.6)	46.0 ± 0.8^F^ (31.3)
	PASS	45.0 ± 13.1 (29.1)	49.0 ± 15.6 (31.8)	19.3 ± 9.7 (50.2)	66.0 ± 14.5^F^ (22.0)	48.1 ± 8.5 (17.7)

^A^ : significant difference with Austria, ^S^: significant difference with Sweden, ^F^: significant difference with France, ^I^: significant difference with Italia (*P* < 0.05). W, win; De, defeat; Dr, draw.

The results presented in Table 3 illustrate the mean and standard deviation for the duration of plays. The typical duration of plays for the two top teams adopting BAL (Austria, *n* = 162, and Sweden, *n* = 157), France (i.e., RUN, *n* = 45), and Italy (i.e., PASS, *n* = 169), was 5.5 ± 1.8, 5.4 ± 1.7, 5.7 ± 2.1, and 5.6 ± 1.6 s, respectively. The overall mean for other European teams (*n* = 570) for duration of plays was 5.3 ± 1.9 s. A statistically significant difference was observed in the duration of plays based on style of play (*p* < 0.05). No significant difference was found between the two top teams, i.e., Austria and Sweden, and France and Italy. The mean playing time of the French and Italian teams was longer than the average duration of plays of the teams from other European countries (*p* < 0.05). There was no significant difference between the Austrian, Swedish, and other European teams. A conformity test indicated a statistically longer plays for all European teams vs. USA teams characterized by RUN style of play (*p* < 0.05). In addition, the mean duration of plays was significantly longer for the French team compared to the PASS and BAL USA teams (*p* < 0.05). Table 3 also presented the average duration of rest based on style for Austrian (*n* = 146), Swedish (*n* = 138), French (*n* = 132), Italian (*n* = 145), and the other European teams (*n* = 465). The rest time between plays exhibited a large standard deviation, but the average duration of rest based on style without extended rest showed a significant difference among styles of play (*p* < 0.05).

**Table 3:** Game statistics.

Play		RUN		PASS		Total bouts	Duration (s) Mean ± SD
Team	Style of play	bouts	Duration (s)	Bouts	Duration (s)		
Austria	BAL	95	5.2 ± 1.4	67	6.0 ± 2.1	162	5.5 ± 1.8
Sweden	BAL	76	5.1 ± 1.4	81	5.7 ± 1.9	157	5.4 ± 1.7
France	RUN	115	5.6 ± 2.2	30	6.1 ± 1.9	145	5.7 ± 2.1
Italy	PASS	58	5.2 ± 1.2	111	5.8 ± 1.7	169	5.6 ± 1.6
Other European teams	BAL	269	5.1 ± 1.5	301	5.3 ± 1.6	570	5.3 ± 1.9^F,I^
	RUN					142	4.8 ± 1.4^A,S,F,I,EU^
NCAA Teams	PASS					145	5.4 ± 1.6^F^
	BAL					136	5.4 ± 1.8^F^
Rest		RUN		PASS		Total bouts	Duration (s) Mean ± SD
Team	Style of play	Bouts	Duration (s)	Bouts	Duration (s)		
Austria	BAL	95	43.8 ± 26.6	51	39.4 ± 16.6	146	42.9 ± 24.8
Sweden	BAL	75	48.1 ± 28.7	63	42.3 ± 20.0	138	45.2 ± 24.6
France	RUN	113	57.4 ± 35.9^A^	19	49.0 ± 29.5	132	55.9 ± 34.7A,S
Italy	PASS	57	55.9 ± 34.1^A^	88	53.6 ± 32.3^A,S^	145	54.5 ± 32.9^A,S^
Other European teams	BAL	240	49.0 ± 52.1	225	43.5 ± 33.3	465	46.7 ± 44.1^F,I^
	RUN					110	46.9 ± 37.5^F,I^
NCAA teams	PASS					108	45.9 ± 24.6^A,F,I^
	BAL					112	47.9 ± 39.1^A,S,F,I,EU^

^A^ : significant difference with Austria, ^S^: significant difference with Sweden, ^F^: significant difference with France, ^I^: significant difference with Italia, ^EU^: significant difference with other European teams (*p* < 0.05). NCCA Teams represents the teams engaged in the National Collegiate Athletic Association (NCAA) Division IA college football championship.

The mean rest duration after a play based on rushing of the offense was shorter for Austria compared to France and Italy (*p* < 0.05). Moreover, the rest interval after a play characterized by passing plays was longer for Italy compared to Austria (*p* < 0.05). Our results indicated a statistically shorter rest duration between plays for BAL vs. RUN and PASS (*p* < 0.05). The mean rest duration between plays of BAL European teams were shorter compared to USA teams, which are characterized by the BAL style of play (*p* < 0.05). Lastly, rest time between plays were longer for France and Italy compared to USA teams, regardless of their style of play (*p* < 0.05).

## Discussion

Based on the study of Bolling et al. (3), the objective of this study was to investigate the effects of team's style of play on the outcome of the U19 European championship. To the best of our knowledge, few published studies have focused on the running-passing activities ratio in American football (12, 13, 16, 23, 25). Our study represents the initial attempt to examine the mean style of play adopted by U19 European teams during an official tournament. Based on the video analysis to determine the rushing style of offense according to the running-passing activities ratio (23), we found that six of the eight teams (i.e., 75.0%) were characterized by BAL style during the U19 European tournament. The other two national teams adopted RUN or PASS style of play: respectively, France, in third place, and Italia, which ranked seventh. The percentage of European teams that adopted a balance style of play was close to the one observed in NCAA Division IA. In fact, Iosia et al. (23) reported that BAL well described the style of play of 88.0% of the top 25 teams according to the coaches poll in 2004 (*n* = 22). The rushing style of offense was considered as running style for only two teams (8.0%). Finally, only one of these top 25 teams adopted a mean style of play that was deemed to be a passing offense during the 2004 season (4.0%<) (23). Thus, the vast majority of youth American football teams tended to adopt a balanced rushing style of offense during the European tournament as in the NCAA Division IA championship for the 2004 season, at least. These first results might lead us to think that the statistics from the NCAA Division could applied to European league. Our findings showed that BAL was the dominant style within the European contest being discussed as the NCAA Division IA championship. Although no study has investigated the effects of playing style on injury, it would seem reasonable to infer that the injury rates would be similar, assuming other conditions are equivalent if the same style of plays was adopted in both continental championship (such as the BAL style mentioned earlier).

However, Bayram et al. (33) observed a marked difference in injuries encountered in another European championship (i.e., United Kingdom) from those reported for US college. This observation could be explained by the quantity and the quality of drives. Indeed, in our present study, the mean number of drives ranged between 9.6 ± 2.5 and 12.3 ± 0.6. The team with a RUN style of play, specifically France, displayed a lower average number of drives with a greater variability between matches. Consequently, no significant impact of style of play was found on the mean drive. The number of plays per drive varied within a narrow range, as illustrated in Table 2 (4.0 ± 19.5–4.7 ± 0.8), which is slightly lower than the average number of plays per drive previously reported (22). The high CV values (>10.0%) across all U19 European teams, regardless of the style of play adopted, suggest an effect of game situation. In the NCAA Division IA championship, Iosia et al. (23) observed a sequence of 11 consecutive plays with a running distance lower than 5 yards per play. They found that the average duration of a run play was 4.86 ± 1.42 s, which is shorter than the estimated mean time for all European teams. The duration of pass plays during the European Championship exceeded that of previous findings (23). The difference between European and American teams may be due to various factors such as tactics, techniques, and decision-making in different game situations. These factors were previously proposed to account for the discrepancy in the mean duration between running and passing plays during the 2004 NCAA Division IA Championship (23). In contrast, our study showed no significant difference in the average duration of pass and run plays. However, the style of play significantly influenced the duration of the game. Overall, a significant difference in the duration of play was observed between RUN, PASS, and BAL teams, with RUN and PASS teams having longer durations than BAL teams. Iosia et al. (23) found a shorter duration of play for RUN vs. PASS and BAL teams, without a significant difference between PASS and BAL. In the present study, the overall average duration of play was not significantly different between RUN and PASS teams, but both were longer than European teams characterized by a balanced style of play. The mean duration of a football play was longer for all European teams compared to RUN USA teams. The average duration of play was longer for the European RUN team compared to the PASS and BAL USA teams. Additionally, significant differences were observed in the rest time between plays between the European and USA teams. Our findings indicate that the style of play had an effect on the duration of rest between run plays and pass plays. As a result, the overall mean rest time was longer for the RUN and PASS European teams compared to the BAL teams. In comparison to the teams that participated in the 2004 NCAA Division IA championship, the average rest time was shorter for the BAL European teams, including the two top teams, while it was longer for the RUN and PASS European teams. Previous research by Iosia et al. (23) suggested that the difference in rest duration was influenced by rules governing play, such as the play clock. Therefore, the combined average play duration and rest between plays suggests different match management between the “top elite” in the U19 category and the European team players. The difference in game duration and recovery time could be responsible for a difference in injury incidence between NCAA Division IA and European championship. This suggests that variations in the amount of playing time and the recovery period between games may influence how frequently injuries occur. Studies have shown that inadequate recovery or excessive game time can increase the risk of injuries (34). For example, shorter recovery periods between games can lead to fatigue, which in turn may contribute to a higher likelihood of musculoskeletal injuries. Similarly, longer or more intense play durations are often associated with a greater risk of overuse injuries, particularly in sports that require repetitive physical exertion, such as American football (35).

American football is a team sport that involves periods of high-intensity activity, with 97–124 plays per match in our study, and can last for more than three hours. During these periods, players are required to perform at a high level of intensity, which demands strength, velocity, and a significant amount of energy (23, 36, 37). However, the short rest periods between plays, which last only 40–50 s, are not enough time for complete ATP-PCR repletion (38). Despite this, there is a positive correlation between ATP-PCR re-synthesis and maximal oxygen consumption in well-trained individuals following single bouts of high-intensity activity (38). The performance of athletes in American football games is reliant on a mix of ATP-PCR and aerobic energy systems. As such, it is essential to focus on the coordination of muscle power and aerobic training. By analyzing video matches based on the running-passing activities ratio, a strength and conditioning program can be developed to support long-term athlete development. Moreover, the video analysis can provide information on the physical and mechanical demands placed on players, which can help prevent hamstring overuse injuries (39, 40). Recently, Wodka-Natkaniec et al. (41) found that injuries to lower limbs accounted for 17.1% and mainly affected the offensive players. Muscle strains were the most frequent type of injury. Significant stress on muscles and joints was particularly observed during running plays. In running plays, there is also a high degree of proximity between players, increasing the risk of injury through both intentional and inadvertent collisions. Hence, activities most commonly associated with injury overall were offensive running play (28.2%) and offensive passing play (10.8%) (42). Previously, Shankar et al. (43) showed that injuries occurred most often during running plays in both practice (40.1%) and competition (62.1%). Running plays were identified as a particular risk factor of concern among high school football players (43). Thus, a systematic video-based ratio of running and passing activities would be beneficial for evaluating the injury risk and developing preventive programs for athlete.

## Limits

The study is based on the methodology of Iosia and Bishop (23), which used standard video cameras at 25 frames per second. The YouTube videos used in this study are also at 25 Hz, which is sufficient for temporal analysis of match sequences but not sufficient to study kicks movements. However, recent literature shows that injuries are mainly related to running phases (42, 43). Finally, we did not take the player's position into account. Makovicka et al. (44) reported that the injuries varied with player position. Running backs and linebackers being the positions most commonly injured (43). This makes sense given their frequent involvement in high-impact plays. the physical demands of certain positions in football place significant stress on muscles and joints, particularly around the hip region. The highest frequency of injuries was however found during running plays. Thus, the systematic video-based ratio of running and passing activities is beneficial for evaluating the physiological and mechanical demands of the game, which can aid in developing strength and conditioning programs for long-term athlete development and injury prevention.

## Conclusion

In the European junior championship, success hinges on two key factors: the ability to adapt one's game style during the tournament, as demonstrated by the Austrian team, and maintaining a consistent number of offensive plays per drive. The systematic video-based ratio of running and passing activities aligns with the “sequence of prevention” model proposed by van Mechelen et al. (2) as a guide for designing sports-related injury prevention programs. Further research on the characteristics of team players may elucidate the discrepancies observed in match management between NCAA Division IA and European U19 teams that implement the same style of play.

## Data Availability

The raw data supporting the conclusions of this article will be made available by the authors, without undue reservation.
